# The impact of midsole hardness on joint angles and plantar loading during running at multiple running velocities

**DOI:** 10.3389/fpubh.2025.1641883

**Published:** 2025-08-06

**Authors:** Luming Yang, Xinye Liu, Yuan Liu, Jing Liu, Shiyang Yan, Guoxia Fei

**Affiliations:** ^1^National Engineering Laboratory for Clean Technology of Leather Manufacture, Sichuan University, Chengdu, China; ^2^Laboratory of Intelligent Clothing & Sport Biomechanics, College of Biomass Science and Engineering, Sichuan University, Chengdu, China; ^3^School of Fashion and Textiles, Hong Kong Polytechnic University, Hong Kong, Hong Kong SAR, China; ^4^State Key Laboratory of Polymer Materials Engineering, Polymer Institute, Sichuan University, Chengdu, China

**Keywords:** gait analysis, speeds, shoes, joint angle, plantar loading pattern, footwear biomechanics, running health

## Abstract

**Introduction:**

Suitable midsoles of running shoes provide better protection for the feet. However, previous studies on the effect of midsole hardness on running biomechanics have ignored the important factor of running velocity and have not reached consistent results. This study set a running velocity with six gradients and aimed to investigate whether the midsole hardness would have a different impact on lower limb joint angles and plantar loading in different velocity ranges.

**Methods:**

Eight male runners ran on a treadmill under 12 conditions (six velocities: self-selected velocity (SSV), 120% SSV, 140% SSV, 160% SSV, 180% SSV, and 200% SSV; two midsole hardness levels: soft and hard). The Noraxon Ultium^®^ insole and Motion IMUs were used to collect data on joint angles and plantar loading. Two-way repeated measures (6 velocities × 2 hardnesses) and paired t-tests were used.

**Results:**

The study showed that at SSV and 120% SSV, the maximum ankle inversion angle in soft midsoles is significantly smaller than in hard midsoles. At 180 and 200% SSV, the maximum hip abduction angle in the swing phase and the maximum force in the metatarsal region in soft midsoles are significantly greater than in hard midsoles, which might lead to a loss of stability and an increased risk of injury in the forefoot. Midsole hardness could have a nonlinear effect on joints and plantar loading as running velocity changed.

**Conclusion:**

The study provided useful information for reducing the potential incidence of running-related injuries based on midsole hardness and running velocity conditions. When considering the impact of midsole hardness on running injury, researchers should pay particular attention to ankle joint motion during 100–120% SSV and hip, ankle joint motion, and metatarsal and arch loading during 180–200% SSV.

## 1 Introduction

Running is a popular and accessible physical activity but is also associated with a high incidence of musculoskeletal pain and injury (1). Wearing the proper running shoes provides better foot protection and reduces the risk of lower limb injuries. In particular, the adjustment of the midsole hardness plays an important role in influencing the risk of running-related injuries (2). The suitable midsole hardness provides good cushioning properties (2), reducing the impact force generated when the heel strikes the ground and reducing local discomfort (2). The design and innovation of the midsole hardness have led to many biomechanical studies, and it has become a hot topic in the fields of sports injury protection and the development of professional sports footwear. It’s also a focal point for research at the intersection of biomechanics and the development of public health interventions. In these studies, the motion of the lower limb joints and plantar loading are meaningful indicators for assessing the risk of running-related injuries from the side (3). Because the joint angle can visually reflect changes in running posture stability (4, 5), foot force can reflect loading patterns and assess the risk of pain (6, 7).

However, previous studies on the effects of different hardnesses on the joint angles and foot load during running were mixed, and no consistent results were reached. Previous studies have shown that at running speeds of 7.2 km/h or 11.8 km/h, the maximum angles of hip and knee flexion were smaller with soft midsoles than with hard midsoles (8), but the range of motion (ROM) of the ankle joint was significantly greater (9), which may increase the risk of ankle sprain in runners. Different studies have found that at a running speed of 10 km/h, there is no difference between hard and soft midsoles in terms of ankle eversion during the stance phase, and the effect of changes in midsole hardness on ankle joint angle is negligible (10). In studies of plantar loading distribution, the overall plantar fascia stress was reduced when midsole hardness was increased at a running speed of 13.7 km/h (11). They concluded that higher midsole hardness supports the arch and helps reduce the risk of plantar injuries. Different studies have found that when the midsole is softer, peak plantar pressure and relative load are reduced (12–14). At the runner’s self-selected velocity for jogging, soft shoes had a lower maximum and average forces in the midfoot region than hard shoes (12). Shoes with cushioning resulted in lower peak pressures in the midfoot and forefoot regions (14), which might reduce the risk of pain in these areas. Overall, the results of the above studies on the effect of midsole hardness on running biomechanics are inconsistent, resulting in weak reliability for runners or researchers to provide insights into the effects of midsole hardness on running potential injuries.

Based on the above studies, the running velocity conditions were not consistent, while the running postures and ground reaction forces were significantly affected by the running velocities (15, 16). Compared with jogging, the maximum hip and knee flexion angle increased by 9.0–25.6^°^ at faster running velocities (15), which could lead to longer stride lengths (17) and exacerbated changes in gait instability (18). When the running velocity increased, the maximum force in the lateral midfoot and metatarsal regions increased significantly by 16.8–47.7% (16), raising the risk of plantar injuries. Meanwhile, the large individual variability in runners’ perception and adaptation of running velocity may have also influenced the results of the study. Therefore, running velocity is an important factor that cannot be ignored when evaluating the impact of midsole hardness on running biomechanics. To date, few studies have systematically accounted for running velocity as a modifier of midsole-hardness effects and explored the implications of midsole hardness and multigradient velocity on running joint angles and plantar loading.

This study set six levels of running velocity to investigate the independent effects of velocity or midsole hardness, or the potential interactive effects between the two, on the lower limb joint motion angles and plantar loading during running, as well as to analyze the gait stability and the potential risk of injuries. We used 3D printing to design running shoes with two different midsole hardnesses. Based on previous reports, we hypothesized that (1) the midsole hardness would have different effects on the motion of the lower limb joints’ angles at various velocity ranges. (2) At faster running velocities, the difference in midsole hardness significantly affects plantar loading.

## 2 Materials and methods

### 2.1 Participants

Rearfoot strike pattern is a common foot strike pattern observed in recreational runners (19). This study selected 12 rearfoot runners from among the 15 healthy participants recruited. The inclusion criteria were as follows: shoe size EUR 43 (male), without significant foot deformities, and having no musculoskeletal or neurological injuries that affected running within the previous 12 months. The final analysis included eight male participants (age: 26.1 ± 4.7 years, height: 174.6 ± 4.8 cm, weight: 67.6 ± 8.3 kg, BMI: 22.1 ± 2.0 kg/m^2^). Before data collection, all participants signed the informed consent form approved by the Sichuan University Ethics Committee (K2025004) and were familiarized with the experimental protocol.

### 2.2 Shoes

Three-dimensional (3D) printing technology is popular in the manufacture of shoe midsoles because it can conveniently develop variable hardness (20, 21). Rhinoceros® 8.0 (Robert McNeel & Assoc, USA) was used to design the midsole structure. The design and adjustment of the lattice structure and void ratio can change the hardness of the shoe midsole and optimize cushioning performance. To combine the requirements of stress dispersion, displacement and energy in the shoe midsole (20), the study selected the Rhombic-dodecahedron structure (R-structure) as the lattice of the midsole. The top and bottom structures connecting the insole and outsole were cut and polished for comfort. The heel thickness of the midsole is 30 *mm*. We controlled the hardness by designing the midsole structure with different void ratios (65 and 70%, respectively) to meet the daily wear requirements of shoes within the mechanical limits of the 3D-printed structure and to achieve the bending hardness requirements of the midsoles (more than 100,000 bending times). The formula for calculating the midsole void ratio is as follows:


$$\text{Midsole void ratio}=\frac{\begin{array}{l} \text{Fully filled volume}-\text{lattice structure}\;\\ \text{volume}\; \end{array}}{\text{Fully filled volume}}$$

**Figure 1:**
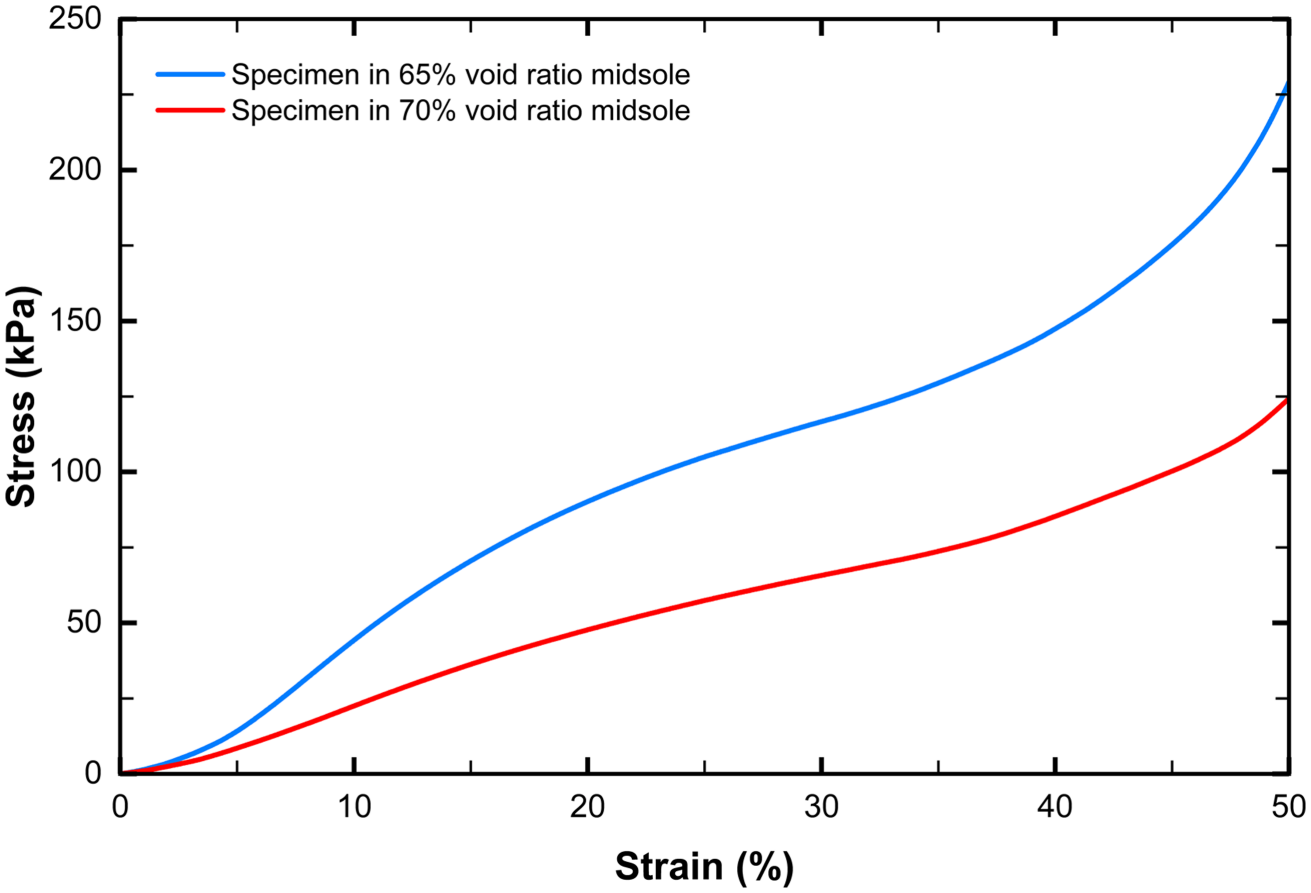
Stress-strain curves of different specimens in compression tests.

Thermoplastic polyurethane (TPU-95A) was selected as the midsole material due to its excellent wear resistance and abrasion resistance, making it particularly suitable for components subject to friction or repetitive motion (22). The selective laser sintering 3D printer (S-480 model, TPM 3D, China) was used to print the midsole. Before printing the two midsoles, a simple study was conducted to characterize the differences in midsole hardness by measuring the compression behavior of the two midsole specimens. Based on the heel height of the midsole, we cut test specimens from the two midsole models in Rhino software, with dimensions of 40 mm (length) × 40 mm (width) × 30 mm (height). The quasi-static uniaxial compression test was conducted using a universal mechanical testing machine (Instron 68TM-30, Instron, USA). The Bluehill Universal software was used to set the test parameters and collect data. Both specimens were compressed at a speed of 10 mm/min until a shortening of 50% was achieved (22). Figure 1 shows the stress–strain curves obtained in the experiment. Both specimens obtained smooth and stable curves. Table 1 shows the results of the compression test and the Shore hardness test. As expected, specimens from the midsole with 65% void ratio had greater structural stiffness (higher modulus and maximum stress).

**Table 1:** Compression test results and hardness of the two types of midsoles.

Midsole type	Elastic modulus (kPa)	Maximum stress (kPa)	Hardness (Shore-C)
A 65% void ratio	1,300	229.3	68
A 75% void ratio	520	124.3	52

After the midsole was fully printed and customized into two pairs of finished shoes, the only difference between the two was the hardness of the midsole. Their hardness is within the range of the midsole hardness of common running shoes on the market (23). The 3D-printed midsole was customized into two pairs of finished shoes, which differed only in the hardness of the midsole. In the subsequent study, they were called soft midsoles and hard midsoles.

### 2.3 Experimental protocol

The Noraxon Ultium® insoles (Noraxon Inc., Scottsdale, AZ, USA) were used to collect plantar loading data. This insole has acceptable accuracy and was used by many researchers to collect data on plantar loading (24). Noraxon Ultium® Motion IMUs (Noraxon Inc., Scottsdale, AZ, USA) were used to simultaneously collect joint angle data during running at a sample rate of 100 Hz. The Noraxon IMU sensors are reliable tools for measuring kinematics and have been used by many researchers to obtain joint angle data (25). According to the definition of anthropometry and Noraxon’s guidelines, sensors were placed on the following locations: pelvis (body area of the sacrum), thigh (midpoint of the lateral femur), shank (front and slightly medial along the tibia medial), foot (upper of the shoe corresponds to the 3–5 metatarsal area). All sensors were secured to Velcro straps around their respective body parts to ensure minimal motion artifacts but were exercised comfortably. Laboratory technicians verify the accuracy of all IMUs throughout the measurement process to ensure that they are properly collected. Participants’ anthropometric data were collected to build the lower extremity biomechanical model in Myo Research 3.20 software. Participants were asked to maintain a neutral reference posture to calibrate IMUs and Ultium insoles (25).

Individuals’ self-selected velocity (SSV) is thought to provide good coordination and stability (26). Participants walked on a treadmill and gradually increased the velocity until reaching their SSV during jogging. The other velocities were set to various increments of the SSV. The mean of the six velocities of the participants was 6.9 ± 0.5 km/h (SSV), 8.3 ± 0.6 km/h (120% SSV), 9.7 ± 0.7 km/h (140% SSV), 11.1 ± 0.8 km/h (160% SSV), 12.5 ± 0.9 km/h (180% SSV), and 13.9 ± 1.0 km/h (200% SSV). Participants wore shoes of two different hardnesses and ran on a treadmill (PK12LT model, RELAX, China) with 0% grade, respectively. Each trial lasted 2 min with a 5-min rest in between. Participants wore two pairs of shoes in a randomized order and had 15 min to familiarize themselves with the running shoes before each test.

### 2.4 Data analysis

Myo Research 3.20 software (Noraxon Inc., Scottsdale, AZ, USA) was used to process plantar loading and joint angle recordings. The IMU biomechanical model that provides joint and segmental kinematics was included in the software and complies with the International Society of Biomechanics recommendations for lower extremity joints (27). The software automatically filters raw data using a robust fusion algorithm (Kalman filter) optimized for IMU data. Data from 20 consecutive running strides were extracted from the middle section of 12 running trials (2 shoes × 6 velocities) for each participant. The gait cycle was defined as the period from one foot strike to the next for the same leg. The software automatically divided the plantar area into four regions (heel, arch, metatarsal and toes) based on eight sensors. The specific sensing distribution is as follows: heel region = average of medial and lateral heel area sensor data; metatarsal region = average of the first metatarsal, third metatarsal, and fifth metatarsal area sensor data; toe region = average of hallux and toe sensors data. Plantar force and joint angle data were time-normalized to 100% of the gait cycle. The gait cycle was divided into stance and swing phases based on plantar force.

The interest variables selected for analysis in this study included maximum angles of hip, knee, and ankle in the sagittal, frontal, and transverse planes, joint ROM, normalized maximum force (NMF), average force percentage (AFP), and percentage of gait cycle subphases. The joint ROM is the absolute value of the maximum angle minus the minimum angle during the gait cycle. The insoles were automatically normalized to each participant’s body weight, and the NMF eliminated the effect of weight differences. The AFP is the percentage of the average force in a certain foot region relative to all areas during the gait cycle. In subsequent studies, it is used to assess gait loading patterns and load transfer in plantar regions. One side (left or right) of their dominant foot was selected (the leg a participant would use to kick a soccer ball) in each participant for data analysis (28).

### 2.5 Statistical analysis

Statistical analysis was performed using SPSS 26.0 (IBM, Armonk, USA). The Shapiro–Wilk test was used to check for normal distribution. A two-way (velocity × midsole hardness) repeated measures ANOVA was used to test for between-group differences in plantar loading, joint angles and joint ROM, and to assess the interactive effect of velocities and midsole stiffness on data. When the homogeneity of variance was rejected, the results were corrected using the Greenhouse–Geisser method. *Post hoc* comparisons at different running velocities for the same midsole hardness were corrected using the Bonferroni method. Paired t-test and Wilcoxon’s signed-rank test were used to compare the kinetic and kinematic data of two midsoles at the same velocity. Confidence intervals for statistical analyses were set at 95% and the significance level was set at *p* < 0.05. Effect sizes (ES) were calculated for all significant mean differences. ES ( $\eta^{2}$ ) assessed ANOVA, interpreted as small (< 0.06), medium (0.06–0.14) and large (> 0.14); ES ( Cohen′d ) assessed t-tests, interpreted as small (< 0.5), medium (0.5–0.8) and large (> 0.8) (29).

## 3 Results

### 3.1 Lower limb joint angles

**Figure 2:**
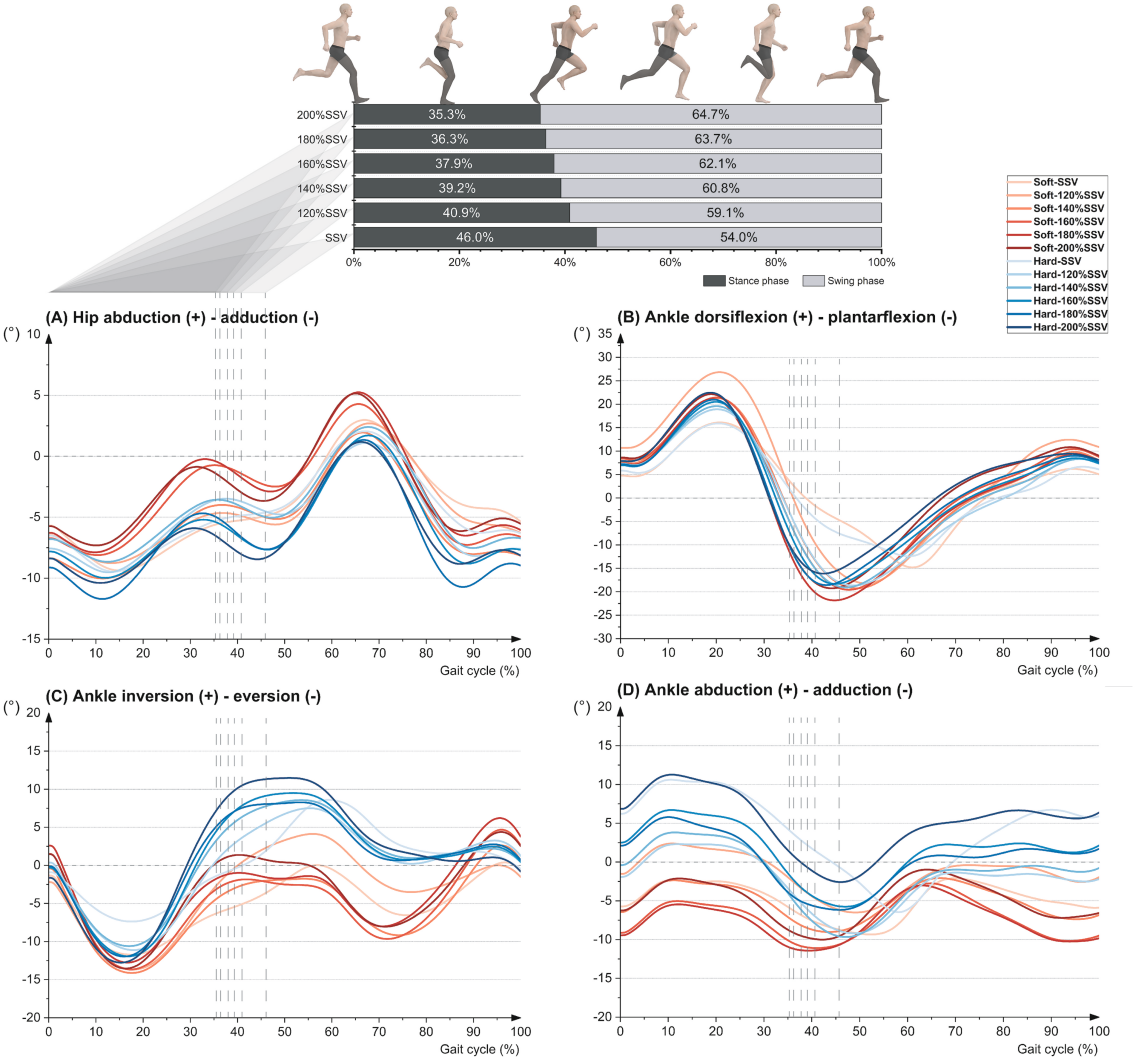
Mean joint angle changes during the gait cycle for running at different speeds in soft and hard midsoles. **(A)** Hip abduction (+) - adduction (−). **(B)** Ankle dorsiflexion (+) - plantarflexion (−). **(C)** Ankle inversion (+) - eversion (−). **(D)** Ankle abduction (+) - adduction (−).

Table 2 shows the *p*-values and ES of the maximum angle and ROM of the lower limb joints affected by velocity and midsole hardness. Table 3 shows the mean and standard deviation of the maximum angle and ROM of the lower limb joints under 12 conditions. (1) When velocity was the main effect, the maximum hip flexion angle of both midsoles and the maximum ankle abduction angle of the hard midsole showed significant changes during the stance phase. In the swing phase, the maximum flexion angles of the hip and knee joints showed significant changes in the two midsoles. During the whole gait cycle, significant changes occurred in the maximum flexion-extension ROM of the hip and knee joints in both midsoles. The maximum abduction-adduction ROM of the knee joint and the maximum plantarflexion-dorsiflexion ROM of the ankle joint in the soft midsole also showed changes. The maximum abduction-adduction ROM of the ankle joint in the hard midsole was changed. (2) When midsole hardness was the main effect, the maximum ankle inversion angle in the stance and swing phases was smaller in soft midsoles than in hard midsoles during SSV and 120% SSV. During 180–200% SSV, the maximum hip abduction angle in the swing phase in the soft midsole was larger than that in the hard midsole. In the 200% SSJV, the soft midsole had a smaller maximum ankle abduction angle during the stance phase than the hard midsole. The soft midsole had a larger maximum plantarflexion-dorsiflexion ROM of the ankle in the gait cycle than the hard midsole. (3) In the interaction effect, velocity and midsole hardness had a significant interactive effect on the maximum inversion and abduction angle of the ankle joint during the stance phase, and the maximum abduction angle of the hip joint during the swing phase. Figure 2 shows the proportion of the stance phase and swing phase of runners’ gait, as well as the motion of lower limb joints during the gait cycle. At the end of the stance phase, the ankle joint has a greater inversion angle in hard midsoles than in soft midsoles.

**Table 2:** The effects (*p*-value, ES) between velocity and midsole hardness on joint maximum angles (^°^) and range of motion (^°^) during running.

Angle type	Velocity		Midsole hardness						Velocity× Hardness
	Soft midsole	Hard midsole	SSV	120% SSV	140% SSV	160% SSV	180% SSV	200% SSV	
MA-Stance phase									
Hip flexion	**0.023, 0.68**	**0.032, 0.66**	0.224	0.357	0.641	0.894	0.409	0.573	0.770
Ankle inversion	0.262	0.766	**0.017, 2.37**	**0.018, 2.14**	0.674	0.401	0.735	0.374	**0.016, 0.78**
Ankle abduction	0.141	**0.017, 0.71**	0.109	0.639	0.603	0.787	0.187	**0.016, 1.38**	**0.043, 0.64**
MA-Swing phase									
Hip flexion	**<0.001, 0.86**	**<0.001, 0.88**	0.244	0.730	0.897	0.496	0.886	0.566	0.267
Hip abduction	0.057	0.602	0.929	0.791	0.409	0.069	**0.044, 1.10**	**0.047, 1.11**	**0.037, 0.19**
Knee flexion	**0.001, 0.84**	**<0.001, 0.89**	0.485	0.344	0.738	0.883	0.599	0.998	0.570
Ankle inversion	0.644	0.565	**0.041, 1.13**	**0.040, 1.14**	0.989	0.320	0.165	0.241	0.504
ROM-Gait cycle									
Hip Flexion-extension	**<0.001, 0.93**	**<0.001, 0.92**	0.152	0.493	0.176	0.133	0.632	0.944	0.292
Knee Flexion-extension	**0.007, 0.79**	**0.005, 0.81**	0.562	0.965	0.616	0.690	0.816	0.766	0.659
Knee abduction-adduction	**0.022, 0.69**	0.218	0.439	0.828	0.480	0.484	0.320	0.122	0.689
Ankle Dorsiflexion-plantarflexion	**0.021, 0.24**	0.396	0.947	0.804	0.203	0.095	0.053	**0.021, 0.75**	0.425
Ankle abduction-adduction	0.374	**0.002, 0.81**	0.328	0.756	0.100	0.060	0.887	0.376	0.135

SSV, self-selected velocity; MA, maximum angles; ROM, range of motion. Bold values: the mean difference is significant at the 0.05 level.

**Table 3:** Means (standard deviations) of maximum joint angles (^°^) and range of motion (^°^) in twelve conditions.

Angle type	Soft midsole						Hard midsole					
	SSV	120% SSV	140% SSV	160% SSV	180% SSV	200% SSV	SSV	120% SSV	140% SSV	160% SSV	180% SSV	200% SSV
MA-Stance phase												
Hip flexion	23.1 (8.3)	20.4 (6.9)	20.1 (8.9)	22.0 (8.3)	25.4 (7.8)	26.4 (7.4)	18.5 (5.9)	17.5 (5.0)	18.4 (5.2)	21.5 (6.6)	22.5 (5.7)	24.6 (5.1)
Ankle inversion	2.2 (1.0)	1.7 (2.8)	2.2 (2.5)	5.6 (6.2)	6.0 (5.6)	5.6 (4.4)	5.8 (1.9)	6.5 (1.5)	6.1 (2.8)	5.1 (1.5)	9.8 (3.5)	8.0 (10.5)
Ankle abduction	4.3 (1.9)	3.8 (1.4)	2.2 (4.6)	3.0 (1.6)	1.1 (3.0)	2.1 (2.3)	3.0 (1.2)	3.4 (1.7)	3.2 (1.9)	2.8 (1.6)	2.9 (1.9)	4.9 (1.7)
MA-Swing phase												
Hip flexion	31.8 (6.8)	30.5 (5.9)	30.5 (8.6)	33.0 (8.7)	38.2 (7.9)	39.7 (6.8)	28.2 (5.0)	29.4 (6.3)	31.0 (8.4)	36.0 (8.3)	38.8 (8.1)	41.9 (8.2)
Hip abduction	3.6 (3.0)	3.3 (3.1)	5.1 (3.9)	6.8 (3.0)	7.5 (3.2)	6.2 (3.4)	3.4 (4.9)	2.9 (3.6)	3.4 (4.1)	2.5 (5.3)	2.8 (5.1)	1.4 (5.1)
Knee flexion	72.9 (11.5)	79.1 (9.4)	80.5 (11.1)	85.6 (11.8)	92.5 (10.6)	93.8 (11.4)	68.8 (11.1)	74.1 (10.9)	78.4 (13.6)	84.5 (16.7)	88.5 (18.0)	93.7 (19.1)
Ankle inversion	6.3 (3.4)	5.3 (2.6)	9.0 (6.6)	9.9 (7.7)	8.5 (3.2)	8.1 (5.5)	9.3 (1.6)	10.7 (6.2)	9.1 (3.2)	15.4 (12.9)	15.5 (13.2)	14.5 (13.7)
ROM- Gait cycle												
Hip Flexion-extension	44.6 (7.6)	41.2 (7.6)	40.7 (14.2)	46.2 (10.7)	53.9 (7.2)	54.3 (7.9)	41.0 (6.2)	40.1 (8.1)	44.7 (10.2)	49.3 (10.7)	52.1 (11.0)	54.4 (9.6)
Knee Flexion-extension	65.4 (2.0)	65.1 (3.6)	67.9 (7.7)	72.1 (8.3)	78.5 (6.8)	79.4 (6.5)	65.7 (4.4)	66.6 (6.1)	71.4 (10.2)	75.6 (12.8)	80.3 (13.6)	83.8 (15.0)
Knee abduction-adduction	13.0 (4.9)	13.0 (4.9)	15.8 (8.3)	17.3 (5.5)	18.7 (6.3)	17.1 (4.1)	11.4 (2.9)	14.1 (9.8)	14.7 (8.9)	16.0 (9.9)	16.0 (7.4)	13.5 (3.5)
Ankle Dorsiflexion-plantarflexion	37.7 (9.6)	40.9 (7.0)	43.3 (8.1)	43.4 (6.0)	43.1 (6.8)	44.4 (6.6)	37.5 (7.7)	39.8 (9.0)	39.7 (7.9)	39.8 (6.6)	39.5 (6.4)	40.6 (6.7)
Ankle abduction-adduction	14.1 (3.6)	15.6 (10.2)	13.7 (4.4)	12.9 (3.3)	16.5 (9.7)	15.6 (5.7)	15.5 (1.0)	17.0 (7.4)	16.4 (2.9)	15.8 (3.6)	17.3 (8.1)	19.9 (8.3)

SSV, self-selected velocity; MA, maximum angles; ROM, range of motion.

### 3.2 Plantar loading

Table 4 shows the *p*-values and ES of NMF and AFP affected by velocity and midsole hardness. Table 5 shows the mean and standard deviation of NMF and AFP under 12 conditions. (1) When velocity was the main effect, the NMF of the metatarsal and heel regions in the soft midsoles changed significantly. The NMF of all regions in hard midsoles changed, except the toe region. The AFP of both midsoles changed in the metatarsal region. (2) When midsole hardness was the main effect, during 120–200% SSV, soft midsoles had greater AFP in the arch region than hard midsoles. At 200% SSV, soft midsoles had greater NMF in the metatarsal region than hard midsoles. (3) In the interaction effects, velocity and midsole hardness had a significant interactive effect on NMF in the metatarsal region and AFP in the arch region.

**Table 4:** The effects (*p*-value, ES) between velocity and midsole hardness on normalized maximum force (%BW) and average force percentage (%) during running.

Plantar regions	Velocity		Midsole hardness						Velocity× Hardness
	Soft midsole	Hard midsole	SSV	120% SSV	140% SSV	160% SSV	180% SSV	200% SSV	
NMF									
Toes	0.167	0.065	0.759	0.570	0.470	0.612	0.795	0.630	0.542
Metatarsal	**<0.001, 0.86**	**0.006, 0.76**	0.334	0.545	0.415	0.487	0.135	**0.032, 0.63**	**0.013, 0.28**
Arch	0.266	**0.038, 0.65**	0.481	0.407	0.751	0.870	0.744	0.822	0.484
Heel	**0.020, 0.70**	**0.009, 0.75**	0.587	0.349	0.387	0.343	0.659	0.396	0.575
AFP									
Toes	0.326	0.825	0.552	0.521	0.176	0.206	0.114	0.118	0.241
Metatarsal	**0.036, 0.65**	**0.028, 0.67**	0.173	0.230	0.134	0.064	0.109	0.061	0.544
Arch	0.347	0.138	0.062	**0.041, 1.13**	**0.018, 1.34**	**0.013, 1.41**	**0.015, 1.39**	**0.014, 1.40**	**0.026, 0.24**
Heel	0.065	0.228	0.924	0.635	0.667	0.534	0.485	0.848	0.599

SSV, self-selected velocity; NMF, normalized maximum force; AFP, average force percentage. Bold values: the mean difference is significant at the 0.05 level.

**Table 5:** Means (standard deviations) of normalized maximum force (%BW) and average force percentage (%) in twelve conditions.

Plantar regions	Soft midsole						Hard midsole					
	SSV	120% SSV	140% SSV	160% SSV	180% SSV	200% SSV	SSV	120% SSV	140% SSV	160% SSV	180% SSV	200% SSV
NMF												
Toes	38.3 (14.6)	39.0 (15.0)	39.0 (14.6)	44.0 (16.5)	45.8 (17.7)	46.9 (16.4)	46.9 (16.4)	36.9 (11.4)	41.4 (14.5)	42.1 (16.0)	46.8 (16.2)	48.5 (18.3)
Metatarsal	72.9 (32.4)	88.5 (26.6)	93.9 (27.9)	99.6 (26.5)	109.5 (28.8)	112.4 (28.6)	79.9 (21.8)	85.1 (21.5)	88.5 (20.9)	94.1 (20.8)	97.7 (19.1)	97.0 (19.1)
Arch	32.4 (12.1)	36.4 (12.0)	38.7 (12.2)	40.4 (12.6)	42.2 (14.2)	43.0 (14.5)	35.2 (18.2)	40.3 (19.4)	40.3 (19.1)	41.3 (23.2)	44.0 (23.8)	44.5 (24.1)
Heel	100.3 (35.8)	94.8 (41.6)	105.1 (51.1)	109.1 (45.2)	113.6 (55.1)	126.0 (55.4)	93.8 (39.0)	79.6 (23.4)	91.3 (23.7)	96.2 (18.1)	106.6 (18.8)	109.0 (20.1)
AFP												
Toes	13.4 (4.0)	15.0 (4.8)	13.5 (3.7)	13.9 (4.0)	13.4 (4.6)	12.8 (4.1)	14.8 (4.4)	16.2 (3.9)	16.6 (4.7)	16.3 (4.6)	16.7 (4.9)	16.9 (5.9)
Metatarsal	36.3 (7.6)	38.3 (8.9)	37.1 (8.7)	36.1 (8.5)	35.9 (9.1)	34.5 (9.4)	41.7 (11.5)	43.5 (10.0)	42.8 (9.1)	43.1 (9.3)	41.4 (7.8)	41.2 (8.1)
Arch	22.2 (8.7)	23.7 (9.5)	24.6 (9.8)	25.1 (10.1)	25.6 (10.3)	25.3 (10.1)	14.9 (5.3)	14.9 (5.5)	14.0 (5.4)	13.2 (6.3)	13.9 (6.0)	13.7 (5.9)
Heel	28.2 (8.1)	23.0 (8.4)	24.8 (7.8)	24.9 (7.3)	25.2 (8.4)	27.3 (8.7)	28.6 (10.8)	25.3 (8.8)	26.7 (7.6)	27.4 (7.1)	28.0 (6.6)	28.2 (7.0)

SSV, self-selected velocity; NMF, normalized maximum force; AFP, average force percentage.

## 4 Discussion

This study investigated the biomechanical response of runners at different velocities with two midsole hardnesses. The results of the study support two hypotheses: (1) midsole hardness has a significant effect on ankle joint and hip joint angle activity at 100–120% SSV and 180–200% SSV. (2) Midsole hardness affects plantar loading patterns. Soft midsoles have greater AFP in the arch region. At 200% SSV, the NMF in the metatarsal region is significantly greater in soft midsoles than for hard midsoles.

The midsole hardness could affect the runner’s posture stability during jogging and fast running. Our study found that velocity and midsole hardness had a significant interactive effect on the maximum ankle inversion angle during the stance phase. Previous studies have found that the impact of running velocity on ankle joint inversion and eversion was not significant (30, 31). In a previous study, the maximum ankle inversion angle during the stance phase while barefoot or near-barefoot was similar to the results of wearing hard shoes in our study (32). Our research showed that as the velocity increased, the maximum ankle inversion angle in the soft midsoles increased, and was similar to that of hard midsoles only at higher running velocities. One possible interpretation is that when running slowly, the stance phase is longer, and the soft midsole is compressed for a longer time and to a larger extent. This might cause the participant to enter the swing phase before the ankle has been inverted. In the late stance, as the ankle is inverted, the oblique and longitudinal midtarsal axes become more skewed, allowing the foot to push off the ground stably and powerfully (33). Wearing soft midsoles results in a smaller ankle inversion angle and a closer-to-parallel axis. This increases the movement of the transverse tarsal joint and increases foot mobility, which could lead to a loss of stability when pushing off (21). This may lead to instability in the runner’s ankles, increasing the risk of sprains, while reduced stability during the push-off phase may compel other joints or muscles to adopt compensatory strategies. The hard midsole might provide a more stable foundation for the foot to enter the swing phase.

In running faster, most of the forward momentum is generated by the swinging rather than the standing leg (34). During the swing phase, our study found that velocity and midsole hardness have a significant interactive effect on the maximum hip abduction angles. The soft midsole had a significantly larger maximum hip abduction angle than the hard midsole at 180% SSV and 200% SSV. At the same time, soft midsoles have a larger ROM in the dorsiflexion-plantarflexion of the ankle than hard midsoles. This could be because runners in soft midsoles need greater joint movement to maintain high muscle activity (35). At the same time, high levels of muscle activity could lead to fatigue, which increases the ROM of joint and further weakens postural stability (28). Our research showed no significant effect of midsole hardness on hip and ankle angle during the stand and swing phase at 140–160% SSV. A previous study has found that the midsole hardness has no significant effect on runners under similar velocities and hardness conditions, and has little relationship with the risk of running injuries (36). Therefore, the effect of midsole hardness on running stability may be more pronounced during slow and high-velocity running.

Previous studies have shown that an increase in running velocity leads to a significant increase in plantar force (16). Hard midsoles often cause higher plantar load (12). Our study found that velocity and midsole hardness had a significant interactive effect on the NMF in the metatarsal region. It is worth noting that as the velocity increased, the NMF in the metatarsal region of the hard midsole with the special 3D-printed structure increased more slowly than that of the soft midsole. At 200% SSV (high-velocity running), the NMF in the metatarsal region of hard midsoles was significantly lower compared to soft midsoles. This is different from previous research (37). One reasonable interpretation could be that the hard midsole based on proper R-structure void ratio differs from ordinary hard materials (20). The R-structure is a unique rhombic structure that satisfies geometric symmetry and constitutes a stable three-dimensional structure. The dense rhombic lattice structure has higher energy absorption and release functions than the sparse structure (38). Under the condition of the midsole with a 65% void ratio based on R-structure, sufficient hardness could provide runners with stable support while offering proper energy absorption and resilience. Compared to a softer midsole, it might significantly reduce the risk of stress injuries in the forefoot region during high-velocity running. Additionally, this study found that midsole hardness affects AFP in the arch region. Hard midsoles had significantly lower AFP in the arch region than soft midsoles. Plantar loading patterns differed between the two midsole hardnesses. During the landing and foot ‘forward rolling’ phases, the hard midsole was rigid enough to provide greater arch support (11), which reduced the AFP in the arch region.

To summarize these results, we have an important finding that the effect of midsole hardness on joint angle and plantar loading during running may be nonlinear and influenced by velocity. At a slow velocity, the vertical force is small, and the soft midsole provides a soft contact between the foot and the shoe, which reduces the compression strain on the foot’s soft tissues (39). The foot has better compliance, thereby reducing the overall plantar loading. When given the same stress, soft midsoles get more compressed than hard ones. As the vertical force keeps going up, the midsole compression will reach its limit when it reaches a certain velocity. The ability of soft midsoles to absorb and disperse plantar force remains stable (39). The ROM of the joint could provide more evidence for this explanation. In our study, the ROM of the soft midsole during ankle dorsiflexion-plantarflexion increased significantly as velocity increased. It remained within a certain range of fluctuation during high-velocity running. A previous study with velocity conditions similar to the 180% SSV in this study found similar results to ours (8). Their study showed a significant effect of midsole hardness on lower limb joint angles in the sagittal plane. The regulatory effect of midsole hardness on lower limb movement control might be amplified during high-velocity running. Overall, the current results show that the impact of different midsole hardnesses on joint angle and plantar load is more pronounced during jogging (100-120%SSV) and high-velocity running (180–200% SSV).

Our study aims to address the mixed results observed in the literature, which might be caused by differences in running surface (40) and shoe type (14), variations between ground and treadmill running (41), and foot strike patterns (42). Our study systematically controlled two key variables and minimized changes in other factors. Running velocity through control of the treadmill conveyor belt (41), at the participant’s SSV and SSV’s incremental velocities. Midsole hardness is achieved through 3D printing, while maintaining consistency in other parts, such as the upper and outsole (40). From the perspective of potential sports injury prevention, our research highlighted the necessity of selecting shoe hardness and running velocity based on runners’ needs, while also providing information for customized running training measures to minimize joint and foot injury burden. Meanwhile, it provided valuable data for two midsole hardness levels within the mechanical range that can be achieved by 3D printing at present. This study has the following strengths and limitations. To the best of our knowledge, this is the first study to investigate the effects of two midsole hardnesses on lower limb joints and plantar loadings across a wide range of relative running velocities. Due to 3D printing limitations, the hardness in this study falls within the range of medium to hard midsoles for commercially available running shoes (23), and the effects of softer midsoles (Shore C-38 to C-51) need to be further investigated. Additionally, due to the restrictions imposed by the inclusion criteria, we ultimately included only eight male participants. Further data collection on female runners is needed in the future.

## 5 Conclusion

Within a wide range of velocity (jogging to fast running), we found for the first time that the effect of midsole hardness on joint angle and plantar loadings during running could be non-linear. Running velocity and midsole hardness had an interactive effect on maximum hip and ankle abduction angles, maximum ankle inversion angles, NMF in the metatarsal region and AFP in the arch region. At 100–120% SSV (jogging) and 180–200% SSV (high-velocity running), soft midsoles might affect the runner’s postural stability. At 180–200% SSV, the hard midsole with special structures allows a flatter rise in plantar maximum force. Compared with soft midsoles, it reduced the maximum force in the metatarsal region. At ranges from 140 to 160% SSV, midsole hardness may not significantly affect the risk of injury to runners in the hip, knee, ankle, and rearfoot and forefoot regions. Therefore, we suggest that a running posture at this range is likely safer, whether wearing a shoe with a soft or hard midsole. These results emphasize that running velocity and midsole hardness should be used together to assess running gait and potential injury risk. This study also provides valuable information for runners and researchers in developing effective running training programs and designing suitable running shoes based on multiple velocities.

## Data Availability

The raw data supporting the conclusions of this article will be made available by the authors, without undue reservation.
